# Rational development of a protective *P. vivax* vaccine evaluated with transgenic rodent parasite challenge models

**DOI:** 10.1038/srep46482

**Published:** 2017-04-18

**Authors:** Ahmed M. Salman, Eduardo Montoya-Díaz, Heather West, Amar Lall, Erwan Atcheson, Cesar Lopez-Camacho, Jai Ramesar, Karolis Bauza, Katharine A. Collins, Florian Brod, Fernando Reis, Leontios Pappas, Lilia González-Cerón, Chris J. Janse, Adrian V. S. Hill, Shahid M. Khan, Arturo Reyes-Sandoval

**Affiliations:** 1 The Jenner Institute, Nuffield Department of Medicine, University of Oxford, The Henry Wellcome Building for Molecular Physiology, Roosevelt Drive, Oxford, OX3 7BN, UK; 2 Leiden Malaria Research Group, Department of Parasitology, Center of Infectious Diseases, Leiden University Medical Center, (LUMC, L4-Q), Albinusdreef 2, 2333 ZA Leiden, The Netherlands; 3 Centro Regional de Investigación en Salud Pública, Instituto Nacional de Salud Pública, 4ta Avenida Norte y Calle 19 Poniente, Tapachula, Chiapas, CP 30740, Mexico; 4 Universidade Federal de Minas Gerais, Belo Horizonte - MG - Brasil

## Abstract

Development of a protective and broadly-acting vaccine against the most widely distributed human malaria parasite, *Plasmodium vivax*, will be a major step towards malaria elimination. However, a *P. vivax* vaccine has remained elusive by the scarcity of pre-clinical models to test protective efficacy and support further clinical trials. In this study, we report the development of a highly protective CSP-based *P. vivax* vaccine, a virus-like particle (VLP) known as Rv21, able to provide 100% sterile protection against a stringent sporozoite challenge in rodent models to malaria, where IgG2a antibodies were associated with protection in absence of detectable PvCSP-specific T cell responses. Additionally, we generated two novel transgenic rodent *P. berghei* parasite lines, where the *P. berghei csp* gene coding sequence has been replaced with either full-length *P. vivax* VK210 or the allelic VK247 *csp* that additionally express GFP-Luciferase. Efficacy of Rv21 surpassed viral-vectored vaccination using ChAd63 and MVA. We show for the first time that a chimeric VK210/247 antigen can elicit high level cross-protection against parasites expressing either CSP allele, which provide accessible and affordable models suitable to support the development of *P. vivax* vaccines candidates. Rv21 is progressing to GMP production and has entered a path towards clinical evaluation.

*Plasmodium vivax* malaria poses a major health risk to 2.85 billion people worldwide living in tropical, sub-tropical and temperate regions^1^. *P. vivax* is the most widely distributed human malaria parasite in the world and considered to be the most difficult to control and eliminate from endemic regions. This is largely due to the parasite’s ability to remain latent in the liver of infected people to subsequently reactivate, weeks to years after an initial infection^2^,^3^.

*Plasmodium* sporozoites enter the blood stream through an infectious mosquito bite and quickly migrate to invade the liver. This ‘pre-erythrocytic’ stage is an attractive vaccine target^4^ used by leading vaccine candidates. This includes the most advanced malaria vaccine RTS,S in the adjuvant AS01. This vaccine aims to prevent a *P. falciparum* infection by stimulating immune responses against the major *Plasmodium* sporozoite surface antigen, circumsporozoite protein (CSP). RTS,S/AS01 has undergone extensive clinical testing and has been shown to induce significant protective immunity against *P. falciparum* in phase III trials^5^. The homologous CSP protein of *P. vivax* (PvCSP) is also actively being investigated as a component of a pre-erythrocytic vaccine against *P. vivax*, with particular emphasis on a vaccine that includes the PvCSP VK210 and VK247 central repeats to protect against the major strains of *P. vivax* circulating worldwide^6^,^7^,^8^,^9^,^10^.

The evaluation of the protective efficacy of *P. falciparum* vaccine candidates and vaccine formulations has greatly benefited from immunization-challenges studies performed in clinical trials using wild-type parasites in controlled human malaria infection (CHMI) studies^6^,^9^,^11^,^12^,^13^,^14^. A similar direct evaluation of vaccines and protective immunity against *P. vivax* in CHMI studies has recently been reported^7^. This has been achieved despite the difficulty of working with *P. vivax*, as this species is difficult to maintain in long-term continuous *in vitro* cultures^15^,^16^,^17^,^18^, and the lack of a safe and effective way to clear latent *P. vivax* forms (hypnozoites) from the liver. While a non-human primate (*Aotus*) challenge model exists, these monkeys are not widely used, due to their limited accessibility, ethical issues and the requirement of splenectomy^19^,^20^. A transgenic rodent *P. berghei* parasite has been generated where the type 1 repeat region of PvCSP has been incorporated into the endogenous *P. berghei* CSP, and these parasites have been used to examine the role of monoclonal and polyclonal antibodies and virus-like particles (VLP)-based immunization in protection against a sporozoite infection in mice^21^,^22^. However, only the repeat region of VK210 has been used and the *P. vivax* CSP epitopes contained in the regions outside of this repeat are absent, thus limiting the use of these parasites in vaccine studies to only those candidates with VK210 epitopes within the repeat region.

Here we report the development of two rodent challenge models suitable to assess the protective efficacy of different PvCSP-based vaccine candidates. In these transgenic *P. berghei* parasite lines, *Pb*CSP has been replaced with full-length *P. vivax* VK210 or VK247 CSP. Sporozoites of these transgenic parasites were used to challenge mice that were previously immunized with various clinically relevant vaccine candidates, expressing or presenting PvCSP that contained regions from VK210 or VK247 or both. An effective approach to generate protective immunity against *P. falciparum* in both animal models and in human volunteers consists of sequential immunization or heterologous ‘prime-boost’ regimes with different viral-vectored vaccines to achieve high frequencies of antigen-specific T-cell responses^23^,^24^. A prime-boost regimen for malaria has been well established in both, mouse and humans, involving an initial ‘prime’ immunization with the chimpanzee adenovirus 63 (ChAd63) expressing a *Plasmodium* antigen followed by a ‘boosting’ immunization with a modified vaccinia virus Ankara (MVA) expressing the same parasite antigen^23^,^24^,^25^. Using this approach, we tested various chimeric forms of PvCSP for efficacy against the transgenic parasites.

We also developed and tested a novel VLP using the Hepatitis B surface antigen platform to present the PvCSP antigen, which we called Rv21. VLPs are known to stimulate B cells to induce high antibody titers; indeed RTS,S/AS01 uses a similar VLP approach^5^, which presents a hybrid polypeptide consisting of a portion of the *P. falciparum* CSP repeat and C-terminal regions fused to the amino terminal end of the hepatitis B virus surface (HepB-S) protein^26^. This VLP has a four-fold excess of HBsAg monomers in the VLP, required to allow expression of the particle in *Saccharomyces* yeast. Here we report an improved VLP, called Rv21 expressed in *Pichia pastoris*. This is based on the recent successful use of the corresponding R21 *P. falciparum* vaccine in pre-clinical (Collins *et al*. submitted) and clinical trials (Venkatraman *et al*. unpublished). Both R21 and Rv21 comprise only the hybrid CS-HBsAg protein in the VLP with no additional HBsAg monomers.

Protective immune responses after vaccination with RTS,S are dependent primarily on antibody responses against the CS central repeat region^27^,^28^. We tested the efficacy of Rv21 in adjuvant vaccination using the new *P. berghei*-PvCSP challenge models in mice. The formulated Rv21 presents both VK210 and 247 repeats and the C-terminal portion of PvCSP on its surface. We show that vaccination with a chimeric VK210/247 antigen induces protective immune responses against sporozoites expressing both the PvCSP VK210 or VK247 alleles. Moreover, we found that an immunization protocol using Rv21 induced complete, sterile protection against a stringent sporozoite challenge, surpassing the efficacy of viral-vectored vaccines expressing a similar PvCSP antigen. This work identifies a highly promising new candidate sporozoite vaccine for *P. vivax* that provides protection against the two major allelic types and highlights the potential and utility of the rodent challenge model to support the development of an efficacious *P. vivax* malaria vaccine.

## Results

### Antigen-specific cellular immune responses are not induced in mice immunized with P. vivax circumsporozoite viral-vectored vaccines (PvCSP vv)

**Figure 1:**
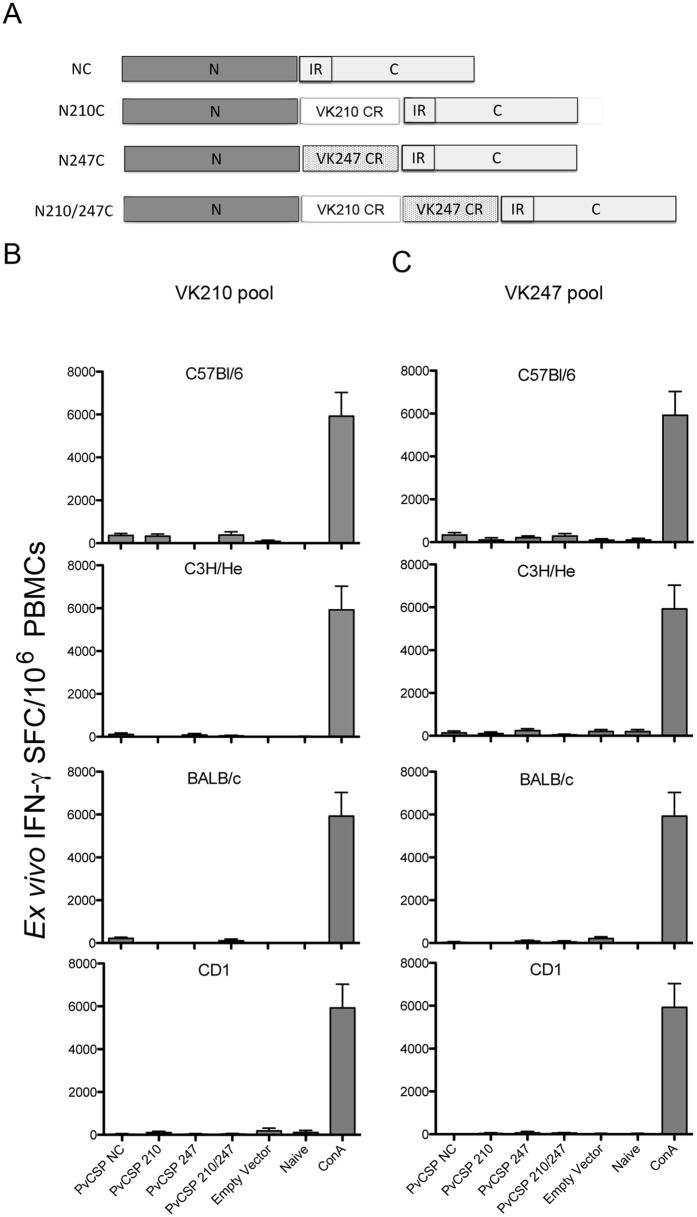
Cellular immune responses following immunization with viral-vectored vaccines (vv; ChAd63 or MVA) expressing different versions of PvCSP. (**A**) Schematic representation of the CSP sequences expressed by ChAd63 and MVA vectors. NC: the amino (N)- and carboxyl-terminal (C)- sequences without central repeat regions (CR); N210C: a fragment containing the amino (N)- and carboxyl-terminal (C)- sequences flanking the VK210 allele; N247C: a fragment containing the amino (N)- and carboxyl-terminal (C)- sequences flanking the VK247 allele; N210/247CR:. a fragment containing the amino (N)- and carboxyl-terminal (C)- sequences flanking a chimeric fragment with both repeat sequences of the VK210 and VK247 central repeats, all contain an insertion region (IR); (**B**) Groups of mice of four strains (C57Bl6, C3H/HE, BALB/c, CD1, n = 6–8) were immunized with different vv using an Ad prime- and MVA boost regimen at an interval of 8 weeks and cellular responses were assessed by *ex vivo* IFN-γ ELISpot on week two after the MVA boost.

We first assessed whether viral-vectored vaccines (vv) expressing the *P. vivax* circumsporozoite protein (PvCSP) could induce antigen-specific cellular immune responses in inbred or outbred mouse strains. We generated four recombinant adenoviruses from the chimpanzee viral-vectored vaccines (ChAd63) and four Modified Vaccinia Ankara (MVA) vv expressing different regions of PvCSP (PvCSP vv). The resulting recombinant viruses contained a series of PvCSP expression cassettes encoding the PvCSP N- and C-terminal sequences of the Salvador I strain of *Plasmodium vivax*, including or excluding the most common central repeat sequences present in the two major PvCSP alleles VK210 and VK247 (Fig. 1A)^8^. First, a set of ChAd63 and MVA vv expressing only the N- and C- terminal sequences and no central repeats were generated (NC); a second set of vv contained a series of the most common VK210 repeats^8^ flanked by the same NC sequences (N210C); a third set expressed VK247 repeats flanked by NC (N247C). Finally we generated a ChAd63/MVA set expressing a chimeric CSP containing both type of repeats (VK210/247) flanked by NC (N210/247C). Groups of mice (n = 6) of four different strains received a homologous ChAd63-MVA prime-boost at an interval of eight weeks between immunizations. Cellular immune responses in mice were quantified two weeks after the final immunization using an *ex vivo* IFN-γ ELISpot assay by stimulating peripheral blood mononuclear cells (PBMCs) with a series of peptide pools designed to assess responses against either N210C or N247C (Fig. 1B,C). An initial experiment using the prime-boost regimen in inbred C57Bl/6 mice did not yield any positive responses (Fig. 1B,C; upper panel). This prompted the assessment of responses in additional mouse strains immunized using the same vaccination scheme. None of these other mouse strains (C3H/He, BALB/c or outbred CD1) showed detectable antigen-specific T cell responses (Fig. 1B,C) and we concluded that the PvCSP molecules that we used in our vv do not have immunodominant T cell epitopes in the animal models tested under our experimental conditions. These results are in agreement with earlier observations using a similar vaccine candidate expressing PvCSP^9^. Nevertheless, splenic follicular T helper cells (Tfh) could be playing a role to support the generation of PvCSP-specific antibody responses and would deserve further exploration.

### Humoral immune responses are induced in mice immunized with the PvCSP vv

**Figure 2:**
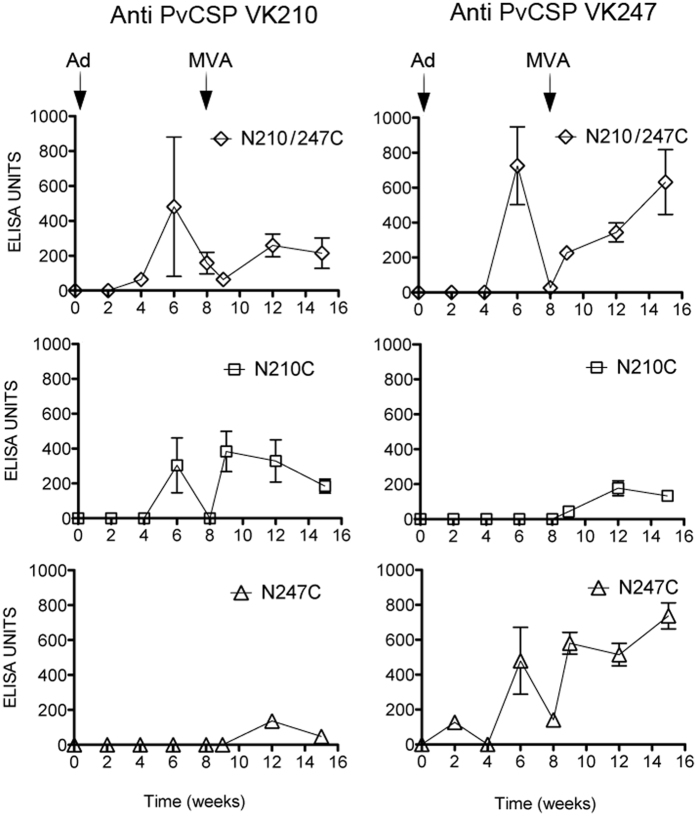
Antibody responses following a prime-boost immunization with viral-vectored (vv) vaccines (ChAd63 followed by MVA) expressing different versions of PvCSP. Outbred CD-1 mice (n = 6) were immunized with vv expressing various versions of PvCSP (N210/N247C, N210C, N247C, NC; see Fig. 1) using a prime-boost interval of 8 weeks. Control groups were mock-vaccinated with vv expressing *P. berghei* CSP and naive mice were injected with PBS, and did not induce any antibodies. Serum was collected every two weeks and antibody titers –total IgG- were quantified using a standardized ELISA against the PvCSP VK210 and VK247 antigens.

We next assessed antibody responses induced by the ChAd63 and MVA vaccination protocols described above, using CD-1 mice. Serum samples were collected fortnightly up until week 16 after the first immunization. We observed that immunization with the vv expressing the chimeric construct N210/247C induced antibody responses to both VK210 and the VK247 PvCSP (Fig. 2, top panel). In contrast responses after an immunization with ChAd63 vv encoding only N210C or N247C, elicited antibodies only against their cognate version of PvCSP (Fig. 2). However, low antibody titres against the heterologous PvCSP allele were detected after a boost with the MVA vv, suggesting that several encounters with the antigen may stimulate low levels of cross-reactive antibody responses, possibly against the conserved N- and C-terminal domains both having a high level of identity of at least 93% in the amino acid sequence. No antibody responses were detected after a ChAd63-MVA immunization with either the NC or with vv expressing rodent *P. berghei* CSP.

### P. vivax sporozoites isolated from Mexico (Tapachula, Chiapas) are recognized by mouse antibodies after PvCSP vv vaccination

**Figure 3:**
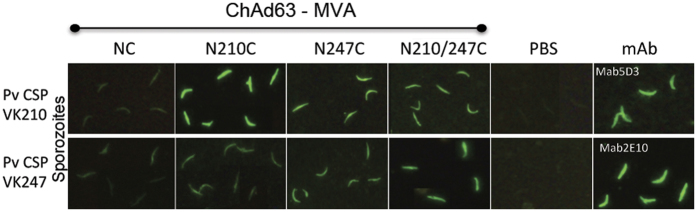
Immunofluorescence Assay (IFA) to assess CSP antibody reactivity of sera from immunized mice on the surface of wild type *P. vivax* parasites isolated from Chiapas, Mexico. Serum obtained from C57Bl/6 mice (n = 6) was incubated with *P. vivax* VK210 (top panel) or VK247 (bottom panel) sporozoites collected from the endemic state of Chiapas, Mexico. Each mouse group was immunized with ChAd63-MVA vv expressing homologous PvCSP transgenes (NC, N210C, N247C, N210/247C) and sera obtained on week two after the final immunization was used at 1:500 dilution. PBS or monoclonal antibodies with specificity to VK210 (Mab5D3, 2.5 μg/ml) or VK247 (Mab2E10, 5 μg/ml) were used as negative or positive controls, respectively. Experiment was made using 6 mice/group and three batches of sporozoites for each sample.

We investigated whether mouse antibodies raised by immunisation with various PvCSP antigens encoded by the vv could recognize CSP on the surface of *P. vivax* sporozoites collected from mosquitoes in the endemic region of Tapachula, Chiapas^29^. We performed immunofluorescence assays (IFAs) using two types of sporozoites in separate slides (e.g. not as a mixing infection), VK210 and VK247. Assays were made using three different sporozoite batches and all yielded similar results. Results shown in Fig. 3 were obtained using sera from C57Bl/6 mice immunized with ChAd63-MVA expressing different regions of the CSP protein as described in Fig. 1a. CD-1 mouse sera yielded similar results (data not shown). Samples were collected at week 2 after the final boost immunization (Fig. 3). Serum from immunized mice reacted with both sporozoites expressing the VK210 allele (Fig. 3 top panel) and sporozoites expressing the VK247 allele (Fig. 3 bottom panel). Mouse sera at 1:500 dilution yielded better results than at 1:100 dilutions and fluorescent pattern was homogeneous with various degrees of intensity: weak, medium and strong. All pre-immune serum and those from primed AdC63 empty vector and boosted with MVA were negative. The monoclonal antibodies 5D3 (from tissue supernatant) and 2E10 (5 μg/ml) were used as positive control for the VK210 and VK247 variants, respectively.

### Development of a rodent challenge model for assessing protective efficacy of the PvCSP vaccination

**Figure 4:**
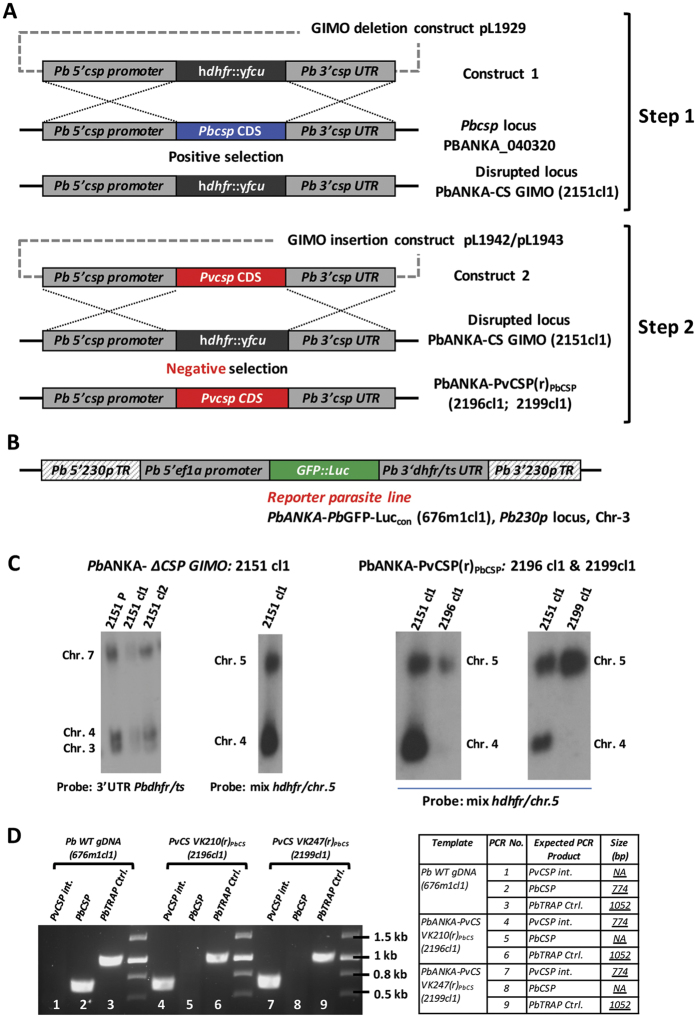
Cloning strategy for the development of two *P. berghei* parasite lines expressing *P. vivax* CSP VK210 or VK247. (**A**) Schematic representation describing the generation of *PbANKA*-*PvCS*P(r)_*PbCSP*_ (2196cl1 and 2199cl1) chimeric lines. The *P. berghei csp (Pbcsp*) gene coding sequence (CDS) was replaced by the GIMO deletion-construct (construct 1; pL1929) using the positive/negative selectable maker (SM; h*dhfr*::y*fcu*) cassette, resulting in the generation of the *Pbcsp* GIMO line (*Pb*ANKA-*PbCSP GIMO*; 2151cl1) after positive selection with pyrimethamine. S*tep 2*: The GIMO insertion-construct (construct 2; pL1942 or pL1943) replaced the SM in the GIMO line with *Pvcsp* VK-210 or VK-247 CDS, respectively, after negative selection using 5-fluorocytosine (5-FC). (**B**) Schematic representation of the reporter *Pb*ANKA parasite line *PbGFP*-*Luc*__*eef1α*__ (676m1cl1), used to generate the replacement gene [RG] chimeric parasites. (**C**) Genotype analysis of Replacement Gene [RG] chimeric parasites and their intermediate GIMO mother-line knock out chimeric parasites using Southern analysis of chromosomes (chrs) separated by pulsed-field gel electrophoresis (PFGE). *Left panel: Pb*ANKA- *ΔCSP GIMO*: 2151 cl1. The correct integration of the *SM* in the right locus in Chr-4 and replacing the endogenous *PbCSP* gene (PBANKA_040320) was confirmed by using the 3′UTR *Pbdhfr/ts* probe. *Right panel: PbANKA*-*PvCSP(r*)__*PbCSP*__: *2196cl1 and 2199cl1*. Confirmation of the correct integration of the *PvCSP* expression construct into the GIMO locus by Southern blot. (**D**) Genotype analysis by diagnostic PCR analysis of chimeric parasite lines to confirm the correct integration of both *PvCSP VK210 and VK247* antigen expression cassettes. Primers sequences used are shown in Table S1, while the expected PCR product sizes and the primer numbers are listed in the table beside the Agarose gel image.

**Figure 5:**
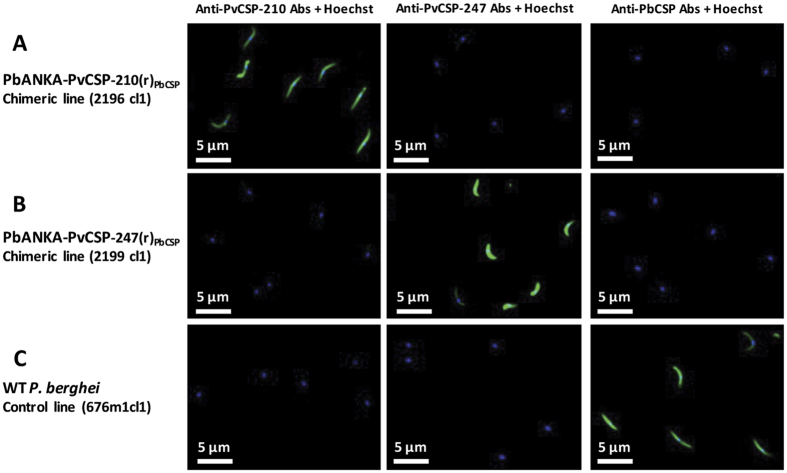
Immunofluorescence assay (IFA) to assess expression of PvCSP on the surface of the transgenic sporozoites. Sporozoites from mosquito salivary-glands were stained with anti-PvCSP-210 (MR4) diluted 200 times, anti-PvCSP-247 (MR4) diluted 200 times, and anti-PbCSP (3D11) diluted 1000 times (3D11 stock (1000X) concentration was at 10 mg/ml and final working concentration was 10 μg/ml). Alexa Fluor^®^ 488-labelled anti-mouse IgG and Hoechst-33342 were also included for the assay. (**A**) Transgenic parasite line 2196cl1 expressing the PvCSP VK210; (**B**) Transgenic parasite line 2199cl1 expressing the PvCSP VK247; (**C**) Wild type *P. berghei* parasite line 676m1cl1 showing expression of PbCSP.

In order to assess the *in vivo* protective efficacy of the *P. vivax* vaccines, we developed rodent challenge models consisting on transgenic *P. berghei* sporozoites where *pbcsp* was replaced with either full-length VK210 *pvcsp* or VK247 *pvcsp*. These transgenic parasites were generated using the gene insertion/marker out (GIMO) based transfection technology^30^,^31^ to generate two Double-step Replacement (DsR) mutants^32^,^33^ that resulted in the coding sequence (CDS) of *P. berghei csp* being replaced with the CDS of the *pvcsp* in a two-step GIMO-transfection procedure (Fig. 4). First, the *pbcsp* coding sequence (CDS) was deleted by replacement with the h*dhfr*::y*fcu* selectable marker cassette (SM) (Fig. 4, Step 1). Subsequently, in these *pbcsp* GIMO-deletion parasites the *pvcsp* CDS was inserted into the same locus thereby replacing h*dhfr*::y*fcu* with the *pvcsp* CDS (Fig. 4, Step 2). These parasites have the full-length *pbcsp* CDS replaced with pv*csp* and were SM free. In the two transgenic parasite lines, PbANKA-PvCS VK210(r)_PbCS_ (line 2196cl1) and PbANKA-PvCS VK247(r)_PbCS_ (line 2199cl1) the *pvcsp* gene is under the control of the *pbcsp* gene promoter (5′UTR) and the transcription terminator (3′UTR) regulatory elements. In addition to expressing PvCSP, these transgenic parasites constitutively express the fusion GFP::luciferase reporter protein, which permits a determination of parasite liver-loads by real-time *in vivo* imaging (Fig. 4B). Correct replacement of the *pbcsp* CDS by the *pvcsp* CDS in the two transgenic lines was confirmed by diagnostic Southern analysis of chromosomes separated by pulsed-field gel electrophoresis and diagnostic PCR on genomic DNA (Fig. 4C,D). Immunofluorescence microscopy of sporozoites using anti-*P. vivax* CSP monoclonal antibodies to the repeat region of the VK210 or VK247 proteins confirmed the expression of either PvCSP VK210 or VK247 in the two transgenic parasite lines (Fig. 5A,B). The *P. berghei* CSP-specific monoclonal antibody 3D11 confirmed expression *P. berghei* CSP in wild-type *P. berghei* sporozoites but anti-*P. berghei* CSP staining was absent in both PbANKA-PvCS VK210(r)_PbCS_ and PbANKA-PvCS VK247(r)_PbCS_ sporozoites (Fig. 5C)^34^,^35^.

**Figure 6:**
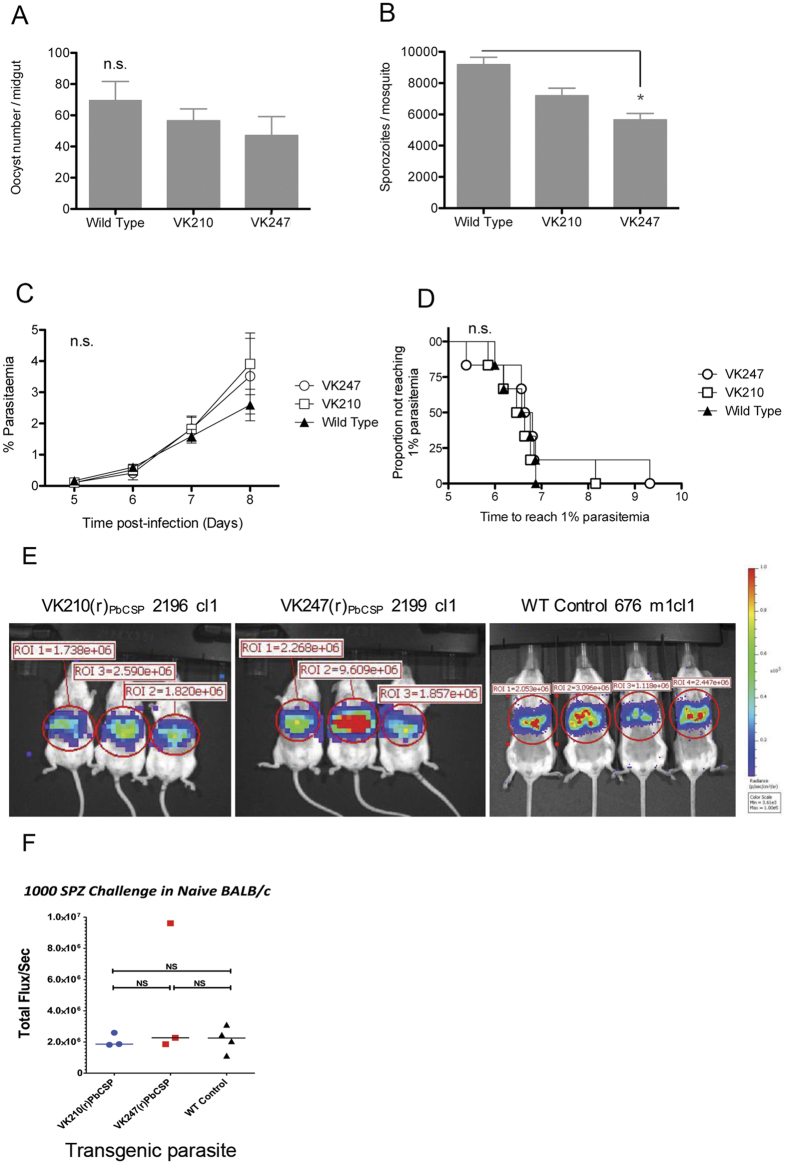
Fitness and phenotype analyses of two transgenic *P. berghei* lines expressing PvCSP VK210 or VK247. Mean of oocysts numbers (**A**) and salivary gland sporozoites (**B**) in *A. stephensi* mosquitoes at day 10 and 21, respectively (3 independent experiments; 40 mosquitoes per experiment; error bars show standard deviation. (**C**) Blood-stage parasitaemia (mean + SEM) in mice after intravenous (i.v.) injection of 2000 sporozoites per mouse. (**D**) The Kaplan-Meier curves illustrate the time to reach 1% parasitaemia (Tto1) in 6 CD1 mice after i.v. injection with 2000 transgenic or wild type sporozoites. Survival analysis was made using a Kaplan-Meier Log-rank (Mantel-Cox) test. n.s.: not significant. (**E**) Parasite liver load in mice upon intravenous injection of transgenic and wild type parasites, determined by *in vivo* imaging of bioluminescence. (**F**) Quantification and comparison of parasite liver load measured as total flux per second.

We next assessed the ability of these two transgenic parasites to produce oocysts and sporozoites since replacing the endogenous *csp* gene with *P. vivax csp* could alter these characteristics. Oocyst production in *Anopheles stephensi* of the two transgenic parasite lines was similar to oocyst production of WT *P. berghei* (Fig. 6A, p = 0.24). Similarly, the PbANKA-PvCS VK210(r)_PbCS_ parasites produced salivary gland sporozoites comparable to WT parasites. In contrast, the PbANKA-PvCS VK247(r)_PbCS_ parasites showed significant (~30%) reduction in sporozoites production compared to WT *P. berghei* parasites (Fig. 6B, p < 0.05). The infectivity of sporozoites of both transgenic parasites was assessed by determination of the pre-patent period (i.e. the time to 1% parasitaemia) after intravenous injection of 2,000 sporozoites in the tail vein of outbred CD-1 mice. All sporozoites were capable of a liver infection and, moreover, the time to 1% parasitemia was not significantly different between WT, PbANKA-PvCS VK210(r)_PbCS_ and PbANKA-PvCS VK247(r)_PbCS_ parasites (6.7, 6.5 and 6.7 days, respectively; Fig. 6C,D). Additional direct evidence to confirm that the transgenic sporozoites are as infectious as WT sporozoites was obtained by measuring parasite liver load or burden at 44 hours after infection using *in vivo* imaging of bioluminescence. Ploemen *et al*. have reported that the analysis of liver infections by whole body imaging shows a good correlation with quantitative RT-PCR analysis of extracted livers^36^. Our *in vivo* imaging results showed no difference in parasite liver loads, i.e. sporozoite infectivity between the transgenic/chimeric lines and wild type parasites (Fig. 6E,F). Therefore, we concluded that the transgenic *P. berghei* parasites expressing PvCSP alleles in place of PbCSP produce fully infectious sporozoites, which are able to complete liver stage development in mice.

### PvCSP central repeat sequences are necessary to induce partial protective efficacy against P. berghei PvCSP transgenic parasites

We next determined vaccination efficacy by challenging vv-immunized mice with the transgenic *P. berghei* PvCSP transgenic parasites. These mice were immunized with the four different (ChAd63-MVA) PvCSP vv, as described above (Fig. 1A). Protective efficacy in these challenged mice was determined by measuring the prepatent period after intravenous injection of 2,000 *P. berghei* PvCSP transgenic sporozoites as described previously^25^.

**Figure 7:**
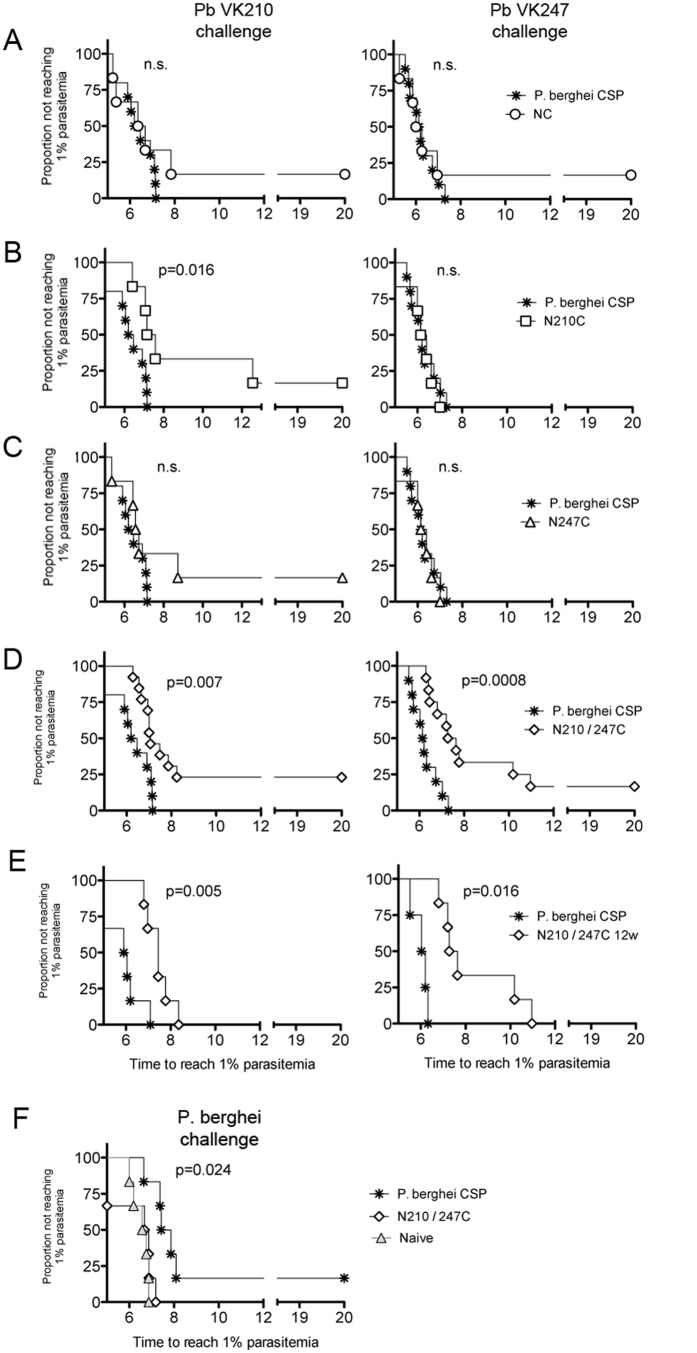
Viral-vectored vaccine (vv) efficacy against a challenge with transgenic *P. berghei* sporozoites expressing *P. vivax* CSP VK210 or VK247. Groups of CD-1 mice (n = 6–10) were immunized with vv expressing PvCSP (N210/247C, N210C, N247C, NC; see Fig. 1) using an Ad prime and MVA-boost regimen. A vv expressing PbCSP was used as control. Two (**A–D**,**F**) or 12 (**E**) weeks after the boost, mice were challenged by intravenous injection of 2,000 transgenic sporozoites expressing PvCSP VK210 or VK247. The Kaplan-Meier curves illustrate the time to 1% parasitaemia (**A**–**F**). As a control PbCSP or N210/247C immunized mice were challenged 2 weeks post-boost with wild type *P. berghei* sporozoites (**F**). Statistical analysis was performed using the Kaplan-Meier survival curves and P values were calculated using a Kaplan-Meier Log-rank (Mantel-Cox) test. Data was obtained from two individual experiments.

Our results indicated that the PvCSP central repeat sequences are necessary to generate protective immunity, as immunisations with NC only (i.e. lacking either VK210 or 247 repeat sequences) failed to induce protective immunity, demonstrated by the prepatent period being similar to mock-immunized mice (Fig. 7A). N210C immunisation resulted in partial protection against a challenge with the transgenic parasites expressing VK210, producing a significant (p = 0.016) prolonged prepatent period. In contrast, N210C immunization did not delay the time to patency of transgenic parasites expressing VK247 (Fig. 7B). Immunisation with the N247C vv did not induce protection against the transgenic parasites expressing either VK247 or VK210 (Fig. 7C). However, immunization with the chimeric N210/247 C induced a significant delay in the prepatent period after challenge with either transgenic parasites expressing either VK210 (p = 0.007) or VK247 (p = 0.0008), while sterile protection in 25% and 16% of mice was achieved after a challenge with VK210 or VK247 transgenic parasites, respectively (Fig. 7D).

While significant protective immunity was maintained 12 weeks after immunisation with the N210/247C vector as determined by a prolonged prepatent period after challenge with either VK210 (p = 0.005) or VK247 (p = 0.016) transgenic sporozoites, durable sterile protection was not induced as all mice eventually developed a blood stage infection (Fig. 7E). We confirmed the absence of unspecific protection induced by N210/247C vv vaccination by challenging mice with wild-type *P. berghei* sporozoites two weeks after boosting immunization. These mice were not protected; protection against wild-type *P. berghei* sporozoites was only induced in mice after a prime-boost (ChAd63-MVA) immunisation regimen with a vv expressing *P. berghei* CS protein (p = 0.024; Fig. 7F). These results demonstrate that immune responses to the central PvCSP repeats are necessary to induce protective immune responses against infection, assuming that N- and C-terminal domains in the NC construct are conformationally correct. Moreover, a chimeric VK210/247 PvCSP vv containing repeats from both forms of PvCSP elicit protective immune responses against sporozoites expressing either VK210 or VK247, which are at least equal to if not better than after immunisation with a vaccine consisting of only one allelic variant of PvCSP.

### Vaccination with the Rv21 VLP induces high levels of protective immunity in mice

The ability to induce antibody-mediated protective immunity using the PvCSP vv and the absence of detectable cellular responses led us to develop a Virus-Like Particle (VLP) based vaccine platform that has a greater potential to generate very potent humoral responses against PvCSP. VLPs are known for their ability to induce remarkably rapid, strong and long-lasting high antibody titres, and are a leading vaccine platform not only for malaria^5^ but also for Human papillomavirus (HPV)^37^. RTS,S/AS01 is based on the hepatitis B surface antigen virus-like particle (VLP) platform, genetically-engineered to include the central repeats and carboxyl (C-) terminus (amino acids 207–395) of the *P. falciparum* CSP antigen^38^. We developed a VLP consisting of the chimeric PvCSP VK210/VK247 central repeats and the CSP C-terminal sequence fused to the Hepatitis B Surface Antigen (HepB-S) gene with a C-terminally placed four amino acid C-tag sequence (Glu-Pro-Glu-Ala). Codon usage of the fusion genes was optimized for expression in *Pichia pastoris* and production of the intracellular fusion protein (PvCSP-HepB-S) was assessed in three protease knockout strains and protease wild-type *P. pastoris* strain using a time course study expression. The double knock-out *P. pastoris* strain for prb1 and pep4 proteases had optimal protein expression levels after 108 hours of methanol induction. The presence of the fusion protein PvCSP-HepB S was confirmed by Western blot analyses using antibodies against PvCSP VK210, PvCSP VK247 and HepB S (Suppl Figure 1A). Presence, size and purity of the Rv21 protein was carried out using a sensitive silver stain technique (Fig. 1A and Suppl Figure 1B).

**Figure 8:**
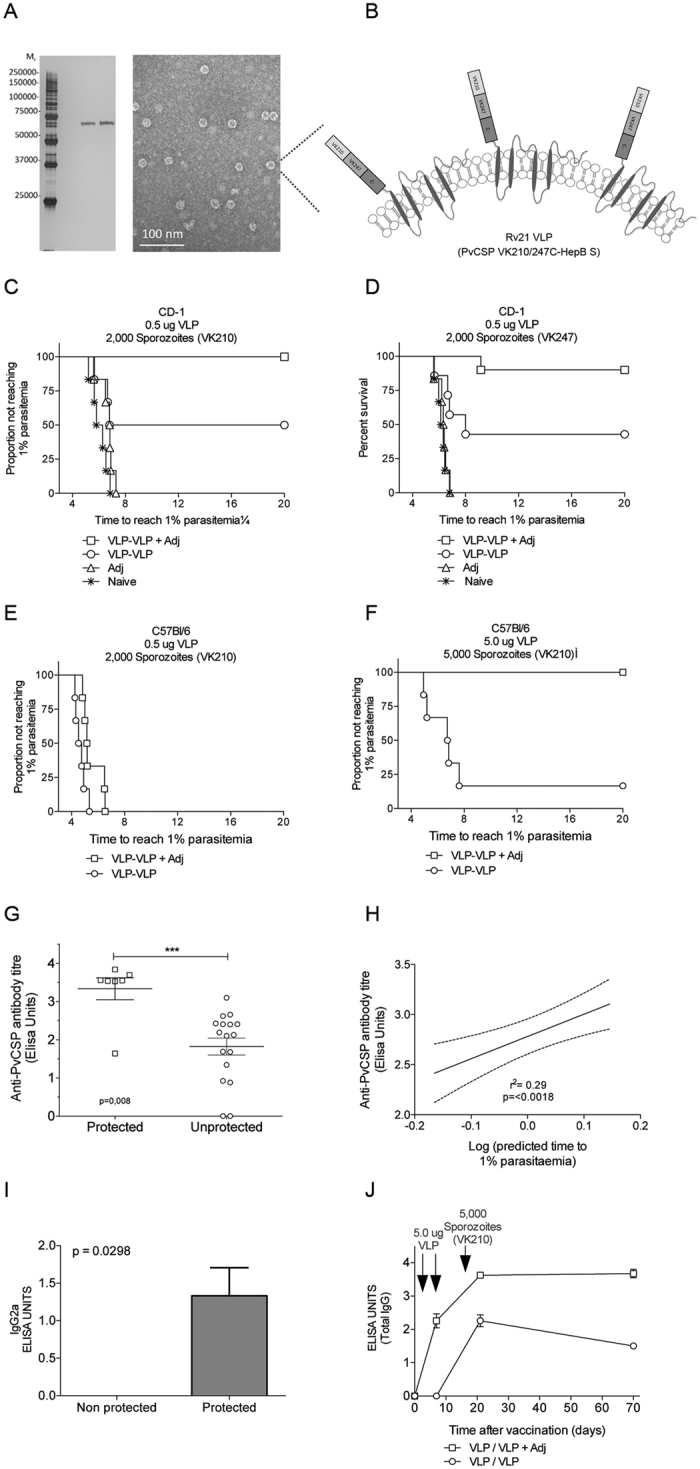
Development, immunogenicity and efficacy of the Rv21 virus-like particle (VLP) presenting the PvCSPVK210/247 antigen fused to the Hepatitis B Surface antigen. The chimeric construct was expressed as a VLP in *P. pastoris*. (**A**) Analysis of protein purity using silver staining after two purification rounds, using size exclusion chromatography of 10 ng of elution sample (**A** and **B**), and visualization of VLPs, by TEM (right panel). (**B**) Diagram showing the proposed structure of Rv21. (**C**) Protective efficacy of Rv21 in CD-1 mice (n = 6) immunized using prime-boost at low doses of 0.5 ug of VLP alone or mixed with Matrix M adjuvant and challenged with transgenic sporozoites expressing *P. vivax* CSP VK210 or (**D**) VK247. Kaplan-Meier curves indicate the time to reach 1% parasitaemia. (**E**) Protective efficacy in C57Bl/6 mice (n = 6) immunized using prime-boost, either a low dose of 0.5 ug or a (**F**) high dose of 5 ug (right) of Rv21 in presence or absence of Matrix M adjuvant (Adj). Challenge of mice vaccinated with 5 ug was increased to 5,000 spz/mouse to increase stringency. Mice were challenged with transgenic sporozoites expressing *P. vivax* CSP VK210 or VK247. The Kaplan-Meier curves illustrate the time to 1% parasitaemia. (**G**) Association and (**H**) correlation of total IgG antibody titers with protective efficacy using data of sera from VK210-challenged C57Bl/6 mice. (**I**) Association of titers of IgG2a with efficacy against pre-erythrocytic malaria. (**J**,**G**) Kinetic of the antibody responses in presence or absence of the Matrix M adjuvant.

The protocol used for purification of the fusion protein VLP has been used for R21 and involved two steps (Collins, Brod *et al*. Scientific Reports, in press). The first consisted of an affinity purification using a capture select C-tag matrix bound to the fusion protein under neutral conditions. In addition to collecting the expected protein band corresponding to the PvCSP-HepB S protein, the sample also contained additional proteins. VLP particle assembly was detected using transmission electron microscopy (TEM) (Suppl Figure 1B). A subsequent purification step was performed by size exclusion chromatography in order to clean up the sample from the other proteins with different molecular size and to remove the high concentration of salts (Elution buffer). A single protein band of expected size of the PvCSP-HepB S protein (75 kDa) was visualized using the silver staining technique and the TEM showed a more homogeneous population of VLPs having a globular shape with some protuberances or spikes on the surface, likely due to the PvCSP protein presence on the surface, as represented in a diagram (Fig. 8A,B). The purified Rv21 was used to immunize mice, using a low dose of 0.5 μg/mouse and employing a homologous prime-boost immunization protocol using an interval of one week between immunizations. Rather than administering the ASO1 adjuvant, standardly used for RTS,S vaccination, Matrix-M adjuvant (Novavax AB Uppsala, Sweden) was used to enhance the immunogenicity and protective efficacy of Rv21. Matrix-M is suitable for human use and consists of saponin-based 40 nm particles that can activate and recruit immune cells to the draining lymph nodes and spleen^39^,^40^. This adjuvant functions by inducing high titres of reactive antibodies supported by a balanced Th1/Th2 response^41^.

The protective efficacy of each prime-boost regimen was assessed by challenging the Rv21-immunized CD-1 mice with a dose of 2000 PbANKA-PvCS VK210(r)_PbCS_ or PbANKA-PvCS VK247(r)_PbCS_ transgenic sporozoites (Fig. 8C,D). Protective efficacy in these immunized mice was determined by measuring the prepatent period as described above. At the low dose (0.5 μg/mouse) of the Matrix-M-adjuvanted Rv21 prime-boost immunization, protective efficacy against challenge with PbANKA-PvCS VK210(r)_PbCS_ sporozoites was 100% and against PbANKA-PvCS VK247(r)_PbCS_ sporozoites 90%, as confirmed by the absence of a blood infection 20 days after challenge (Fig. 8C,D). In contrast, all mice in the naïve group developed blood stage parasitemia with a prepatent period of 4–5 days. In the absence of the Matrix-M adjuvant, immunisation of mice using the VLP homologous prime-boost regime still generated sterile immunity in 50% of the VLP-immunized mice challenged with PbANKA-PvCS VK210(r)_PbCS_ sporozoites (Fig. 8C) and 43% of immunized mice were protected against a challenge of PbANKA-PvCS VK247(r)_PbCS_ sporozoites (Fig. 8D). These results indicated the importance of both the VLP and the adjuvant in inducing protective immune responses with this vaccination regime. In addition, our results showed that PvCSP VLP-based vaccination induced higher protective efficacy than the viral-vectored vaccine candidates. ELISA analyses of antibody responses to vaccination supported the importance of Matrix-M adjuvant to elicit high antibody titres to PvCSP, even after a single (priming) immunisation with Rv21 (Suppl Figure 2A,B). A prime-boost regimen with Rv21 in the absence of the Matrix-M adjuvant-induced higher titres than those by a single vaccination but still lower compared to the prime-boost regime with the adjuvant. We concluded that the Rv21 antibody responses benefit from the co-administration of an adjuvant, both in a single immunization or a prime-boost regime. Importantly, the antibody titres induced by two doses of Rv21 in matrix-M were over 100-fold higher than those induced by the viral vectored prime-boost regime (Fig. 2 and Suppl Fig 2A).

We tested Rv21 efficacy in C57BL/6, a mouse strain that has previously shown to be highly sensitive to a *P. berghei* sporozoite infection and for which we have previously failed to induce sterile immunity using a *P. vivax* TRAP vaccine candidate^42^. Immunisation of mice with a Rv21 dose as described above (0.5 μg) followed by a challenge with 2,000 transgenic PbANKA-PvCS VK210(r)_PbCS_ sporozoites failed to show protective efficacy (Fig. 8E). However, a 10x increase in the Rv21 dose (5 μg) showed, for the first time in our hands, complete protection in C57BL/6 mice even against a challenge with a higher dose of 5,000 PbANKA-PvCS VK210(r)_PbCS_ sporozoites (Fig. 8F). Antibody titres measured as total IgG were associated with protection (Fig. 8G,H) and from these, only IgG2a was statistically associated with protection after challenging C57Bl/6 mice (Fig. 8H), while none of the other isotypes, such as IgM, IgG1, IgG2b and IgG3 showed any statistical association with protection (Suppl Figure 2D–F). Rv21 immunization (in Matrix M) induced antibody titres that remained consistently high for up to 70 days (Fig. 8J), while the inclusion of Matrix-M adjuvant enhanced antibody avidity with the time, as the addition of urea decreased antibody titres by 26.2% when using adjuvant and 100% in its absence (Suppl Figure 2C).

Our results indicate that immunization with a chimeric VK210/247 vaccine can induce immunity to the two major strains of *P. vivax*, based on the type of PvCSP central repeats. Moreover, high levels of protective immunity levels can be achieved using a VLP platform in the presence of the Matrix-M adjuvant, even in a mouse strain that is difficult to protect against a sporozoite infection.

## Discussion

The circumsporozoite protein (CSP) is a leading vaccine target for both *P. falciparum* and *P. vivax* vaccines^26^,^43^. For an effective vaccine based on *P. vivax* PvCSP it has been suggested that immunity must be generated against the two major forms of PvCSP that are circulating around the world^8^,^10^,^11^, encoded by the VK210 and VK247 alleles, thereby generating protective immunity against the majority of *P. vivax* parasites circulating worldwide. While cellular responses play a role in protective immunity against *P. falciparum* liver infections^23^,^24^,^25^, the induction of strong humoral responses against both *P. falciparum* and *P. vivax* appears to be critical to the induction of protective immunity after immunization with vaccines based on CSP^6^,^9^,^12^. High and long-lasting IgG titers have been reported using a variety of platforms including bacterial-expressed recombinant PvCSP, a hybrid CSP molecule as a polypeptide or PvCSP expressed by the chimpanzee adenovirus ChAd63 and a chimeric circumsporozoite protein^6^,^11^,^12^. The VK210 central repeat region of *Pv*CSP has been expressed as a virus-like particle (VLP) within the loop of the hepatitis B virus core antigen, and both the VLP and a recombinant multi-epitope polypeptide rPvCSP-ME elicit long-lasting antibody titers of high intensity and avidity^6^. Recently, targeting the CSP antigen of *P. vivax* with a recombinant soluble protein VMP001 and the particulate antigen CSV-S,S have shown to induce robust humoral and cellular immune responses, especially with the particulate CSV-S,S vaccine^10^, that induced high levels of sterile protection in *Aotus nancymaae* monkeys, and for which an association between antibodies to the central repeat region and protection was observed^12^.

In this study we describe the induction of humoral responses against PvCSP in the absence of detectable *ex vivo* IFN-γ antigen-specific T cell-mediated immunity, when using either viral vectored (vv) or virus-like particles (VLP)-based vaccine platforms encoding the VK210 and/or VK247 PvCSP repeat regions. Importantly, we demonstrate that antibodies generated after either a VK210 or VK247 based vv-immunization are able to recognize native *Pv*CSP on the surface of *P. vivax* sporozoites isolated in the field.

In the absence of immune correlates of protection, assessment of the protective efficacy of *P. falciparum* malaria vaccine candidates has relied on determining protective efficacy directly against a parasite infection in phase IIb field studies or in controlled human malaria infection (CHMI) trials^44^,^45^. Due to technical challenges, which include the lack of *P. vivax in vitro* blood-stage cultivation system, evaluating protective efficacy of *P. vivax* candidate antigens in CHMI is difficult. Consequently, evaluation of the protective immunity of *P. vivax* pre-erythrocytic vaccines is more dependent on preclinical evaluation in animal models before proceeding with phase I and II trials. Preclinical evaluation of *P. vivax* pre-erythrocytic vaccine candidates has been performed in rodents^6^,^9^,^11^,^21^,^42^,^46^ and non-human primates^10^,^12^. However, pre-clinical evaluation of protective efficacy in monkeys presents additional difficulties, which include their restricted access, as well as the need to conduct these studies only in regions where both, monkeys and *P. vivax* parasites coincide^19^,^20^. These limitations in evaluating vaccine efficacy very much reduce the rate at which different *P. vivax* vaccine candidates are ranked and evaluated, and therefore assessed in clinical trials.

In order to evaluate the protective immunogenicity of our PvCSP vaccine candidates and to rationally prioritize vaccine candidates for further clinical development, we developed two novel *in vivo* challenge models in rodents. We generated two transgenic *P. berghei* parasite lines in which *P. berghei* CSP has been replaced with full-length *P. vivax* VK210 or VK247 CSP. The use of transgenic *P. berghei* parasites expressing *P. vivax* or *P. falciparum* antigens is a practical and affordable option, which can be used in laboratories with access to mouse models. Indeed, such models have been previously used to analyze protective immunity has been reported for several *Plasmodium* vaccine antigen candidates such as TRAP, CSP and a transmission-blocking vaccine (TBV)^21^,^22^,^31^,^47^. For example, transgenic *P. berghei* parasites expressing different forms of *P. vivax* CSP have been generated by Espinosa *et al*.^21^ and Mizutani (2014) and used to analyze protective immunity upon vaccination. The first study used transgenic parasites expressing a chimeric CSP protein consisting of sequences of both *P. berghei* and *P. vivax*, whereas the latter study used transgenic parasites expressing the *P. vivax* CSP corresponding to amino acids His_24_ to Asp_351_ of the PvCSP(Sal) gene minus its signal sequence and glycosylphosphatidylinositol (GPI) anchor. As mentioned, it has been suggested that for *P. vivax* CSP-based vaccines conferring protection against the 2 major isoforms of PvCSP (VK210 and VK247) alleles would be immunologically optimal to recognize and confer protection against most *P. vivax* parasites circulating worldwide^11^. However, confirmation of this hypothesis has remained elusive in the absence of challenge experiments using parasites that express either the VK210 or the V247 allele. We therefore decided to replace the complete coding sequence of *P. berghei* with the coding sequence of the two major alleles of PvCSP. Interestingly, PvCSP appears to be able to complement the function of PbCSP in the transgenic parasites, both in the mosquito and in liver infection. The VK210-expressing parasite produced normal numbers of sporozoites similar to a wild type infection. However, while VK247-expressing sporozoites also exhibited wild type hepatic-infectivity this line produced 30% fewer sporozoites, suggesting that the exact sequence of the CSP repeat region influences sporozoite formation.

The normal *in vivo* infectivity of the transgenic PvCSP *P. berghei* sporozoites make these transgenic parasites excellent tools to better analyze protective immune responses induced by novel vaccine platforms that induce immunity against PvCSP, and in turn can be used to prioritize vaccine candidates for clinical assessment. Using these parasites we were able to confirm that epitopes present within the repeat regions are required to confer cross-protection against parasites expressing either the VK247 or the VK210 Pv*csp* allele. Consequently, we used these transgenic parasites to compare different PvCSP vaccination approaches, specifically, ChAd63/MVA viral vectored vaccination and a novel *Pichia pastoris*-expressed Hepatitis B Surface Antigen fusion protein VLP, Rv21. While both approaches were able to generate antibody responses against PvCSP, immunization with Rv21 surpassed antibody responses by viral vectors and demonstrated very high levels of protective immunity, even in hitherto difficult to protect C57BL/6 mice. Prime-boost Rv21 vaccination was able to confer 100% and 90% sterile immunity to CD-1 mice challenged with a dose of 2000 VK210 or VK247 expressing transgenic *P. berghei* sporozoites, respectively. Recently, Whitacre *et al*.^22^ have also shown high protective efficacy in C57BL6 mice using an *E. coli* derived VLP that expressed VK210 repeat from PvCSP, this VLP-platform was based on the woodchuck hepatitis virus core antigen (WHcAg). However in order to generate protective immunity they required very high doses (100 μg) of the VLP^22^. When Rv21 vaccination was combined with Matrix-M as an adjuvant we were able to induce very strong protective immunity at VLP doses as low as 0.5 μg or 5 μg in CD-1 or C57Bl/6 mice, respectively. We consider that the use of C57Bl/6 mice is an optimal mouse strain to support vaccine efficacy studies through sporozoite challenge experiments, compared to CD-1 mice that are less suited for these studies. This adjuvant has been successfully and safely used in humans, most recently for *P. falciparum* malaria (Venkatraman *et al*. unpublished), and consists of immune-stimulating complexes (ISCOMs) in the form of 40 nm particles made with adjuvant-active saponins (*Quillaja saponaria*), cholesterol and phospholipids^41^. Matrix-M has been shown to enhance immune responses in humans when combined with influenza or West Nile vaccines^39^,^48^. The observations on the strong adjuvanting properties of Matrix M upon Rv21 is of particular interest as VLPs based on the HepB Surface antigen have previously been considered less immunogenic than those using the HepB core antigen^22^. Our study shows that HepB-S based VLPs can be highly immunogenic and protective, even at low doses between 0.5 and 5 μg. Future studies should address the contribution of the N- and C- termini sequences to the protective efficacy of VLP vaccines such as Rv21 and CSV-S,S, the latter containing the N-terminus repeat.

In conclusion, the newly developed transgenic *P. berghei* parasites expressing PvCSP alleles VK210 and VK247 repeats have been highly effective tools that have helped develop and optimize vaccine candidate platforms targeting PvCSP. They have confirmed the value of using immunization strategies that induce immune responses against the two major isoforms of PvCSP and show that they can greatly improve protection against infection. Moreover, using this testing system we have been able to evaluate a novel VLP-based vaccine candidate, Rv21, which consists of a chimeric VK210/247 *Pv*CSP fusion molecule combined with hepatitis B surface antigen, and have demonstrated that this vaccine is highly efficacious, conferring high levels of protective immunity even in mice that have previously been difficult to protect using another leading *P. vivax* vaccine candidate, TRAP^42^. Importantly, to our knowledge, this is the first evidence of protection against both sporozoites expressing both isoforms of the PvCS, VK210 and VK247. Rv21 has now received support for GMP production and has entered a path towards clinical trials.

## Materials and Methods

### Animals

Female inbred BALB/c (H-2^d^), C57BL/6 (H-2^b^) and outbred CD1 (ICR) mice were used for the assessment of immunogenicity and protection after challenge. Tuck-ordinary (TO) outbred mice were used for parasite production and transmission. Mice were purchased from Harlan (UK). Transgenic parasites were developed in Leiden University Medical Centre (LUMC) using 6-week old Swiss mice (Charles River).

### Immunization of mice

Viral vectors: Mice were primed with simian adenoviral vector 63 (ChAd63) encoding PvCSP at a dose of 1 × 10^8^ infectious units (iu) and 8 weeks later boosted with vaccinia-modified virus strain Ankara (MVA) encoding the same transgene at a concentration of 1 × 10^6^ plaque forming unit (pfu) per mouse. All viral vector vaccines were administered intramuscularly (i.m.) in endotoxin-free PBS and no adjuvant was used for vv immunizations.

Rv21 VLP vaccine: Rv21 was administered in endotoxin-fee PBS mixed with 12 μg of Matrix M Adjuvant (Isconova, Sweden, now Novavax, MD, USA). The Rv21 vaccine was injected intramuscularly at a dose of 0.5 μg/mouse in CD1 mice, while C57Bl/6 mice received two doses, 0.5 μg or 5 ug/mouse in different experiments. The vaccination regimen for Rv21 consisted of homologous prime-boost at an interval of one week between immunizations.

### Ethics statement

All animals and procedures were used in accordance with the terms of the UK Home Office Animals Act Project License. Procedures were approved by the University of Oxford Animal Care and Ethical Review Committee (PPL 30/2414). Animal experiments performed at LUMC were approved by the Animal Experiments Committee of the Leiden University Medical Center (DEC 12042). The Dutch Experiments on Animals Act was established under European guidelines (EU directive no. 86/609/EEC regarding the Protection of Animals used for Experimental and Other Scientific Purposes).

### Parasite production

Wild type and transgenic parasites used to challenge mice were produced at the insectary of the Jenner Institute. Female *Anopheles stephensi* mosquitoes were fed on infected TO mice. Briefly, exflagellation was first confirmed and mosquitoes were exposed to anaesthetized infected mice for 10 minutes. Mosquitoes were then maintained for 21 days in a humidified incubator at a temperature of 19–21 **°**C on a 12 hour day-night cycle and fed with a fructose/PABA solution.

### Parasites

The wild type (WT) reference line cl15cy1 of *P. berghei* ANKA^49^ and the reporter *Pb*ANKA parasite line *Pb*GFP-Luc_con_ (676m1cl1). *Pb*GFP-Luc_con_ parasite expresses a fusion protein of GFP (mutant3) and firefly luciferase (LUC-IAV) under the constitutive *eef1a* promoter and is SM free^49^. The reporter-cassette is integrated into the neutral *230p* locus (PBANKA_030600). For details of *Pb*GFP-Luc_con_, see RMgmDB entry #29 (http://www.pberghei.eu/index.php?rmgm=29).

### Generation of DNA constructs and genotyping of the transgenic parasites

To generate the transgenic parasites where the *P. berghei csp* gene (PBANKA_040320) coding sequence (CDS) has been replaced by the CDS of *P. vivax csp* (PVX_119355), we used a 2-step GIMO transfection protocol^31^,^50^. In the first step we deleted the *P. berghei csp* CDS and replaced it with the positive-negative selectable marker, to create a *P. berghei csp* deletion GIMO line (PbANKA-CSP GIMO). In order to this we generated pL1929 construct that is based on the standard GIMO DNA construct pL0034^50^. This construct contains the positive-negative (h*dhfr*::y*fcu*) selection marker (SM) cassette, and was used to insert both the *Pbcsp* 5′ and 3′ gene targeting regions (TR), encompassing the full-length promoter and transcription terminators sequences respectively. The linear pL1929 DNA construct was introduced into *Pb*GFP-Luc_con_ parasites using standard methods transfection^49^. Transfected parasites were selected in mice by applying positive selection by providing pyrimethamine in the drinking water^49^. Transfected parasites were cloned by limiting dilution^51^, resulting in the PbANKA-CSP GIMO line (2151cl1). Correct deletion of the *P. berghei csp* CDS was confirmed by diagnostic PCR-analysis on gDNA and Southern analysis of pulsed field gel (PFG) separated chromosomes as described^49^. Primers used for PCR genotyping are listed in Table S1.

In the second step we replaced the positive-negative SM in the PbANKA-CSP GIMO genome with the CDS of either *P. vivax* VK210 or VK247 *csp* by GIMO transfection to create the two *P. berghei* transgenic CSP replacement lines. This was achieved by modifying the construct used in the first step (pL1929); specifically, the h*dfhr*::y*fcu* SM cassette was removed and replaced with *P. vivax csp* CDS sequence. The *P. vivax csp* CDS was ordered from GeneArt (Regensburg, Germany) (i.e. VK210) or cDNA (VK247) Both the *P. vivax* VK210 and VK247 CSP constructs (pL1942 and pL1943, respectively) were sequenced to ensure there were no mutations in the *P. vivax csp* CDS. These constructs were linearized using SacI and PacI restriction enzymes outside of the 5′ and 3′ TRs before transfection. These constructs were used to transfect parasites of the PbANKA-CSP GIMO line (2151cl1) using standard methods of GIMO-transfection^30^. Transfected parasites were selected in mice by applying negative selection by providing 5-fluorocytosine (5-FC) in the drinking water of mice^52^. Negative selection results in selection of chimeric parasites where the h*dhfr*::y*fcu* SM in the *csp* locus of PbANKA-CSP GIMO line is replaced by the CDS of *P. vivax* CSP Selected transgenic parasites were cloned by the method of limiting dilution^51^. Correct integration of the constructs into the genome of chimeric parasites was analysed by diagnostic PCR-analysis on gDNA and Southern analysis of pulsed field gel (PFG) separated chromosomes as described^49^. Primers used for PCR genotyping are listed in Table S1. This method creates transgenic ‘gene replacement’ *P. berghei* parasites that do not contain *P. berghei csp* gene CDS but express either *P. vivax* VK210 (PbANKA-PvCSP VK210(r)_PbCS_; 2196cl1) or VK247 *csp* (PbANKA-PvCSP VK247(r)_PbCS_; 2199cl1) under the control of the *P. berghei csp* regulatory sequences.

### Phenotyping of reporter and transgenic parasites

The growth of blood stages of the reporter and transgenic *P. berghei* parasites was determined during the cloning period as described^30^,^49^. Feeding of An. *stephensi* mosquitoes, determination of oocyst production, sporozoite collection were performed as described^30^. Expression of *PvCSP*-*VK210 and PvCSP*-*VK247* antigens in sporozoites of the transgenic parasites was analysed by immunofluorescence- staining assay (IFA), using anti-*P. vivax* antigen monoclonal antibodies (anti-*Pv*CSP-VK210 (MR4) or anti-PvCSP-247 (MR4) antibodies; diluted 200 times) or anti-*Pb*CSP 3D11 antibodies as a control; diluted 1000 times. Purified sporozoites were fixed with 4% paraformaldehyde in PBS for 20 min on ice, then washed three times with PBS and blocked with 20 ul10% FCS + 1% BSA in PBS for 30 min at room temperature. The excess blocking medium was removed, followed by the addition of 20–25 uL primary monoclonal antibody in 10% FCS + 1% BSA in PBS (blocking medium) for 1–2 hours at room temperature or overnight at 4 °*C*. After incubation the primary antibody was removed and the slides washed three times with PBS, followed by the staining with the secondary antibody (Alexa Fluor^®^ 488 Goat Anti-Mouse IgG from life technologies, Cat# A-11001) diluted 800 times in 10% FCS + 1% BSA in PBS (blocking medium) for 1 hour at room temperature. After washing three times with PBS, nuclei were stained with 2% Hoechst-33342 (Cell Signaling Technology #4082S) in PBS for 10 minutes at room temperature, washed twice with PBS and left to air-dry, this followed by adding Fluorescence Mounting Medium (Dako, code S3023) before complete dry out. Cover slips were mounted onto the slides, and the slides were sealed with nail polish and left to dry overnight in dark. The parasites in both blue and green channels were analyzed using a DMI-300B Leica fluorescence microscope and images processed using ImageJ software.

### Immunofluorescence Assay for Wild-Type P. vivax isolates from Mexico

Antibody recognition to native, wild-type *P. vivax* isolates from Mexico was assessed by IFA using a technique described earlier^53^. Briefly, sporozoites were produced by infection of laboratory-reared *An. albimanus* mosquitoes that fed on blood from patients infected with *P. vivax*. Mosquitoes were maintained for 15–18 days and sporozoites were collected by dissection of the salivary glands and deposited in multiwell IFAT slides at a concentration of 2,000 sporozoites/well to make individual slides containing *P. vivax* sporozoites VK210 or slides with only VK247, keeping them separate and not as mixed parasites. Confirmation of the VK210 and VK247 variant types was made as described earlier^54^ using primers to amplify the repeat VK210 or VK247 regions followed by slot blot and hybridization to ^32^P-labeled oligonucleotide probes for VK210 and VK247 and a final confirmation was made using the monoclonal antibodies 5D3 and 2E10, specific for the VK210 and VK247 variant types. Slides were kept frozen at −70 °C until used. The assay was performed using sera from C57Bl/6 and CD-1 mice, which was incubated with three different batches each of sporozoites, yielding similar results between both strains. Air-dried sporozoites were also incubated with anti-VK210 (monoclonal antibody 5D3 at 2.5 μg/ml) or anti-VK247 (monoclonal antibody 2E10 at 5 μg/ml) monoclonal antibodies as positive controls^29^. Slides were analyzed using a confocal microscope and titers were calculated using the highest dilution that gave positive fluorescence.

*Efficacy studies: Determination of liver parasite liver load by real*-*time imaging and determination prepatent period (after challenge of immunized mice with transgenic sporozoites*)

To determine the efficacy of the liver-stage vaccines, transgenic *P. berghei* infected *A. stephensi* mosquitoes were dissected 21 days post-feed and salivary gland sporozoites resuspended in RPMI-1640 media (Sigma-Aldrich). 2000 sporozoites in 100 μl were injected i.v. into the tail vein per mouse, into both vaccinated and naïve controls.

### P. vivax CSP DNA sequences

Four versions of ChAd63 and four of MVA were designed and produced to express various versions of the *P. vivax* CSP protein. One consisted of only the N- and C- terminal regions (NC); a second viral vector expressed VK210 repeats inserted in between the N- and C-terminal sequences (N210C). A third vector expressed VK247 inserted between the N- and C-terminal sequences (N247C) and a final design consisted on a chimeric VK210/247 flanked by similar N- and C-terminal repeats (sequences detailed below).

DNA transgenes were synthesized by GeneArt (Regensburg, Germany) and constructs were previously modified to improve antigen expression within the hosts cells, modifications included codon optimization for mammalian use and replacement of the endogenous PvCSP leading sequence for tPA (human plasminogen activator) (GenBank Accession no. K03021). In addition, the transmembrane GPI-anchor domain was removed from the *Plasmodium vivax* circumsporozoite protein (PvCSP) genes to allow protein secretion from any virus-transduced cell. The chimeric PvCSP vaccine insert consisted on the N- and C- terminal region from the Salvador I strain (NCBI Reference Sequence XP_001613068.1) flanking the central repeat regions of the circumsporozoite (CSP) VK210 of the Belem strain (GenBank accession number P08677) or VK247 of the Papua New Guinea (PNG) (GenBank accession number M69059.1). The central repeat region was designed using segments of 9 amino acid repeats that are most frequently found in *Plasmodium vivax* as follows: VK210 (5x(GDRAAGQPA), 4x(GDRADGQPA), 1x(GNGAGGQAA)) and VK247 (2x(ANGAGNQPG/ANGAGGQAA), 1x(ANGAGDQPG/ANGAGDQPG), 1x(ANGADDQPG/ANGAGDQPG), 1x(EDGAGNQPG/ANGAGDQPG))^8^. A region II-plus was included in the C-terminal sequence of the vaccine (EWTPCSVTCG)^55^, as well as an insertion region in the C-terminal sequence of PvCSP (GAGGQAAGGNA)^56^. The VK210 vaccine insert consisted on the Salvador I strain sequence, while the VK247 consisted of the PNG sequence with the accession numbers mentioned above. A control viral vector lacking the expression of any transgene was used as a control in mock-vaccinated mice.

### Viral vector construction

All of DNA constructs required for ChAd63 were cloned in two steps. In the first step, unique Acc65I and NotI sites were used to insert the synthetic transgenes into an adenovirus entry plasmid. The transgene was placed upstream of BGH poly(A) transcription termination sequence and under the control of the long cytomegalovirus (CMV) promoter (containing a regulatory element, an enhancer and an intron A). The entry plasmid also contained attachment L (attL) sequences, which were required for site-specific recombination with attachment R (attR) sites located on the destination vector.

In the second step of cloning, an *in vitro* Gateway reaction was performed mediated by LR Clonase II system (Invitrogen), whereby the transgene of the entry vector was integrated into the destination plasmid by site-specific recombinase through an attL-attR interaction. The diagnostic PCR was performed to confirm the desired integration before completing the production of the recombinant ChAd63.

Similarly, all of the malarial genes were inserted into an MVA shuttle vector using a similar cloning strategy. Unique Acc65I and XhoI sites were used for transgene restriction and ligation and the transgenes were inserted under the control of an endogenous P7.5 promoter. PCR and RFLP were used to verify the correct insertion before linearization of the MVA plasmid.

### Rv21 HepB Surface antigen VLP design and development

A gene containing a chimeric CSP sequence comprising the repeat regions VK210 and VK247, followed by the C-terminal sequence (210/247C) was designed to be fused to the Hepatitis B surface antigen (HepBsAg) followed C-terminally by the peptide EPEA (C-Tag) for affinity purification. The sequence was codon-optimised for optimal expression in yeast and purchased from GeneArt^®^ (InVitrogen). DNA sequences were similar to those expressed by recombinant ChAd and MVA viruses, but without the N-terminal CSP region. The construct was cloned into the pPink-HC intracellular Pichia plasmid and amplified in *E. coli*. Upon plasmid linearization, four strains of *Pichia pastoris* (knock out for ade2, ade2-pep4, ade2-prb1, ade2-pep4-prb1) were electroporated and white colonies were selected using PAD selection plates. Cultures for protein expression were grown in glycerol containing medium and expression was induced by addition of methanol. Growth kinetics were analysed to determine conditions for optimal protein production The VLPs were purified from yeast lysates by C-Tag affinity chromatography followed by size exclusion chromatography. (Collins *et al*. manuscript in preparation). Fractions from gel filtration were analyzed by SDS-PAGE and subsequently transferred to nitrocellulose membranes, blocked in 5% skimmed milk/PBS followed by addition of primary and secondary antibodies. VLP-containing fractions from the gel filtration column were detected with anti-HBsAg, anti-PvCSP VK210 and anti-PvCSP VK247 primary mouse antibodies, diluted in 3% BSA/PBS to 1:200, 1:20,000 and 1:20,000 respectively (MR4). Silver staining was used to analyse purity of the fractions from the FPLC column and of progressive stages of the VLP purification process. The samples were run on Mini-PROTEAN 12% gels (BioRad) with the Pierce unstained protein ladder (Thermo Scientific) run for reference. The gels were subjected to silver staining using a Pierce silver stain kit (Thermo Scientific). Direct staining of gels was carried out according to manufacturer’s instructions. The particle-containing fraction from the sephacryl gel filtration column was negatively stained with 2% uranyl acetate as follows: the Rv21 sample was imaged using a FEI Tecnai 12 Transmission Electron Microscope (TEM). The TEM protocol used to evaluate the different Rv21 vaccine purification steps used a FEI Tecnai 12 Transmission Electron Microscope (TEM) at an accelerating voltage of 80 kV. The samples (10 μg) were absorbed for 1 min to a glow-discharged C-Formvar coated Cu-grids (300 mesh) and negatively stained with 2% (w/v) uranyl acetate, pH 4.5. The VLPs were visualized taking under 20,000× magnification, and the photographic records were performed on 4 Megapixel Gatan Ultrascan™ 1000 CCD camera.

### Whole IgG Enzyme-linked immunosorbent assay (ELISA)

ELISAs measuring total IgG were carried out as described previously^24^. Serum antibody endpoint titers were taken as the x-axis intercept of the dilution curve at an absorbance value three standard deviations greater than the OD405 for serum from a naïve mouse. Results were also calculated using a standardized ELISA by including a high-titre reference serum from hyper-immune mice to each ELISA plate to produce a standard curve, which in turn was used to quantify and assign ELISA units to each sample^57^. Briefly, Nunc Maxisorp Immuno ELISA plates were coated with the antigens diluted in PBS to a final concentration of 1 μg/mL. Each plate contained a standard curve, negative and positive controls, as well as serum samples of mice pre-boost (1:300) or post-boost (1:3000). An anti-mouse IgG coupled to alkaline phosphatase was used as a secondary antibody. Development was made with 4-nitrophenylphosphate diluted in diethanolamine buffer. Data was fitted to a four parameter hyperbolic curve^58^.

For avidity ELISAs, standard curve ELISAs were performed as described above except following 2 h incubation of serum at room temperature plates were washed 6 times with PBS/Tween and 100 μl 7 M urea added to serum wells for 10 min. Plates were then washed 6 times with PBS/Tween and the standard curve ELISA carried out as before. Avidity index was calculated as the percentage of log_10_ EU of urea-treated compared untreated serum samples, following the procedure described by Grangeot-Keros, L. *et al*.^59^.

For IgG subclass ELISAs, standard curve ELISAs were performed as described above, using the Biorad Mouse Typer Sub-Isotyping kit with these modifications (protocol for mouse typer sub-isotyping kit, Biorad): after serum incubation subclass-specific rabbit anti-mouse IgG was applied at 50 μl per well in triplicate. The standard curve was incubated with kappa-chain specific IgG. After 1 h incubation plates were washed 6 times with PBS/Tween and goat anti-rabbit horseradish peroxidase conjugate applied (50 μl/well, 1:3000). After 1 h plates were washed 6 times with PBS/Tween and developed for 14 min using 100 μl/well peroxidase substrate solution (Biorad). Reactions were stopped by adding 100 μl/well 2% oxalic acid.

### Expression and purification of PvCSP VK210 and VK247 proteins

The *P. vivax* protein CSP210 (Belem; GenBank accession number P08677) and the protein CSP247 (PNG; GenBank accession number M69059) were produced as previously described^60^. In addition, a chimeric CSP protein containing the repeats from both PvCSP210 and PvCSP247 was produced. Briefly, a codon-optimized synthetic gene (chimeric CSP210/247) was synthesized by GeneArt^TM^. CSP210/247 was subcloned into the expresion plasmid PHLsec (His_6_ tag), which is driven by the chicken β-actin/rabbit β-globin hybrid promoter^61^. The resulting PHLsec CSP210/247 was verified by DNA sequencing.

All secreted proteins were purified from media supernatants from PEI-transfected HEK-293T cells using roller bottles (2,125 cm^2^) at 37 C and 5% CO_2_, using immobilised metal ion affinity chromatography (IMAC) medium (resin) precharged with nickel ions (Ni Sepharose™ excel, GE Healthcare). Conditioned media was filtered through a 0.45 μm membrane (Millipore). IMAC purification was performed using a distilled water wash step with 5 column volumes (CV) at a flow velocity of 100 cm/h, equilibration step with 5CV of equilibration buffer (20 mM sodium phosphate, 0.5 M NaCl, pH 7.4/flow velocity: 150 cm/h), load sample step (flow velocity: 150 cm/h), wash step with 20 CV of wash buffer (20 mM sodium phosphate, 0.5 M NaCl, 20 mM imidazole, pH 7.4/flow velocity: 150 cm/h), linear elution step with 2 CV of 7% elution buffer (20 mM sodium phosphate, 0.5 M NaCl, 500 mM imidazole, pH 7.4/flow velocity: 150 cm/h) and 2 CV of 70% elution buffer (flow velocity: 150 cm/h). Elution samples after IMAC purification were analysed using a 12.5% SDS-PAGE under reducing conditions and proteins were visualized with Silver stain and Western Blot analyses using the monoclonal antibody anti PvCSP-VK 210 and VK247. (Obtained through MR4 BEI Resources NIAID, NIH: Monoclonal Antibody Sporozoite ELISA Kit, Anti-*Plasmodium vivax* Circumsporozoite Proteins, MRA-1028K, contributed by R. A. Wirtz). Samples were concentrated using an Amicon^®^ Ultra centrifugal filter system (Life Technologies) until reaching 10 ml of final volume. Removal of possible contaminant proteins and salts was done by size exclusion purification (SEC). SDS-PAGE electrophoresis and subsequent Coomassie and Silver staining was performed to assess protein purity.

### Peptides

Crude 20-mer peptides overlapping by 10 amino acids and representing full-lengths of *P. vivax* CSP VK210 and VK247 were synthesized by Mimotopes (Victoria, Australia). Individual peptide pools were used at a final concentration of 5 μg/mL per peptide.

### *Ex*-*vivo* IFN-γ ELISPOT assay

*Ex vivo* IFN-gamma (IFN-γ) ELISPOTs were carried out using PBMCs isolated from the blood as previously described^62^,^63^. The assay was performed using MAIP ELISPOT plates (Millipore) were where PBMCs (1 × 106 cells/well) were co-cultured with splenocytes (2.5 × 10^5^ cells/well) in Minimum Essential Medium Eagle supplemented with 1% L-Glutamine, 10% Foetal Calf Serum (FCS), Penicillin and Streptomycin (100 UI/ml). Overlapping peptide libraries consisting on 20-mer peptides overlapping by 10 were purchased at PEPSCAN Presto, The Netherlands. Peptides spanned the whole sequence of the P. vivax CSP VK210 and VK247 transgenes and were used to stimulate the co-cultures for 16 hours. DMSO, phytohemagglutinin (10 U/mL) and medium controls were used in the same assay plate. Anti-mouse IFN-γ mAb and development reagents were used according to the manufacturer specifications (Mabtech). Spots were counted using an ELISPOT counter (Autoimmun Diagnostika (AID), Germany). Results are expressed as IFN-γ spot-forming units (SFU) per million PBMCs.

### Infection of mice

The CD-1 and C57Bl/6 mice that were vaccinated with 0.5 μg of Rv21 were challenged with 2,000 wild type or transgenic *P. berghei* sporozoites. When C57Bl/6 mice were vaccinated with increased doses of 5 μg/mouse of Rv21, a higher dose of 5,000 sporozoites per mouse was used for a challenge. Infection was monitored from day 5 to 20 by Giemsa staining of blood smears.

### Statistical model for parasitaemia prediction

Percent parasitaemia was used to calculate the time required to reach a blood-stage infection of 1% or time to 1% parasitaemia. This was predicted using a linear regression model as described previously^25^. Briefly, blood parasite counts were obtained for 3–5 consecutive days starting on day 5 after the challenge. Blood smears were stained with Giemsa and percentages of parasitaemia calculated in all animals. The logarithm to base 10 of the calculated percentage of parasitaemia was plotted against the time after challenge and Prism 5 for Mac OS X (GraphPad software) statistical analysis package used for generating a linear regression model on the linear part of the blood-stage growth curve.

### *In vivo* imaging after malaria sporozoite challenge

Bioluminescent luciferase signal was quantified by imaging the whole animals using the *in vivo* IVIS 200 imaging system (Caliper Life Sciences, USA), as described elsewhere^36^,^64^,^65^. Briefly, 44 hrs after the intravenous injection with 1000 transgenic *P. berghei* sporozoites the mice were anaesthetized in batches of three using isofluorane, their fronts shaved and D-luciferin (Synchem Laborgemeinschaft OHG, Germany) injected into the neck at a concentration of 100 mg/kg in sterile PBS (Sigma, US). Animals were imaged for 120 seconds at binning value of 8 and FVO of 12.8 cm, 8 minutes after the injection of D-luciferin. Mice were kept anaesthetized throughout the whole procedure. Quantification of bioluminescence signal was performed using Living Image 4.2 software (Caliper Life Sciences, USA). The region of interest (ROI) were set around the liver area of the mouse body and kept constant for all of the animals. The measurements were expressed as a total flux of photons per second of imaging time.

### Statistical analysis

For all statistical analyses, GraphPad Prism version 5.0 for Max OS was used unless indicated otherwise. Prior to statistical analysis to compare two or more populations, the Kolmogorov-Smirnov test for normality was used to determine whether the values followed a Gaussian distribution. An unpaired t-test was employed to compare two normally distributed groups, whereas Mann-Whitney rank test was used for comparing two non-parametric groups. If more than two groups were present non-parametric data was compared using Kruskal-Wallis test with Dunn’s multiple comparison post-test, whereas normally distributed data were analyzed by one-way ANOVA with Bonferroni’s multiple comparison post-test. The effect of two variables was explored using two-way ANOVA with Bonferroni’s multiple comparison post-test. Correlation strength was tested using either Pearson’s or Spearman’s tests as indicated in the results chapters. Kaplan-Meier survival curves were used to represent protective efficacy to a challenge with any *P. berghei* parasite lines. All ELISA titres were also log10 transformed before analysis. The value of p < 0.05 was considered statistically significant (*p < 0.05, **p < 0.01, **p < 0.001, and ***p < 0.001).

## Additional Information

**How to cite this article**: Salman, A. M. *et al*. Rational development of a protective *P. vivax* vaccine evaluated with transgenic rodent parasite challenge models. *Sci. Rep.* **7**, 46482; doi: 10.1038/srep46482 (2017).

**Publisher's note:** Springer Nature remains neutral with regard to jurisdictional claims in published maps and institutional affiliations.

## Supplementary Material

Supplementary Information
