# Supplementary material for: Rational development of a protective P. vivax vaccine evaluated with transgenic rodent parasite challenge models
Source: Sci Rep. 2017 Apr 18;7:46482. doi: 10.1038/srep46482 (PMC5394459; doi:10.1038/srep46482)

Supplementary Information

Rational development of a highly protective *P. vivax* vaccine evaluated using transgenic rodent parasite challenge models

Ahmed M. Salman[1,2*], Eduardo Montoya-Díaz[1*], Heather West[1*], Amar Lall[1*], Erwan Atcheson[1], Cesar Lopez-Camacho[1], Jai Ramesar[2], Karolis Bauza[1], Katharine Collins[1], Florian Brod[1], Fernando Reis[4], Leontios Pappas[1], Lilia González-Cerón[3], Chris J. Janse[2], Adrian V. S. Hill[1], Shahid M. Khan[2] and Arturo Reyes-Sandoval*[1].

Supplementary Information

Supplementary Table S1. Primers used for PCR genotyping and expected PCR product sizes.

Primer name	Primer sequence[1]	Expected PCR product	PCR size (bp)
PvCSP-trans (F6)	TGACATGCATATGTGTTGGTTG/	*PvCSP 5' int.*	*744*
PvCSP-trans (R5)	GCTGATTGTCCAACATGTGC		
1054	CCAAAGGAACTTAAACGAGCTATG/	*PbCSP*	*744*
1055	CTTATACCAGAACCACATGTTACG		
7393	ATCGACTAGTAAAGCCTCGCTACG/	*PbTRAP Control*	*1052*
7394	GTGAGTCAGATGGACTTTCTGGTAG		

[1] **The primer sequences (i.e. Forward/Reverse) surrounding the regulatory region are shown with the restriction site used (bold and underlined) to introduce the region into the transfection construct.**

Supplementary Figure 1. Development, of the Rv21 virus-like particle (VLP) presenting the PvCSPVK210/247 antigen fused to the Hepatitis B Surface antigen.

The chimeric Rv21 construct was expressed as a VLP in *P. pastoris* (A) Western blot to confirm expression of the VK210, VK247 and HepB surface antigen (S Ag) after purification (100 ng of elution sample. (B) Analysis of protein purity using silver staining after affinity purification of 10 ng of the elution sample and visualization of VLPs by transmission electron microscopy (TEM).

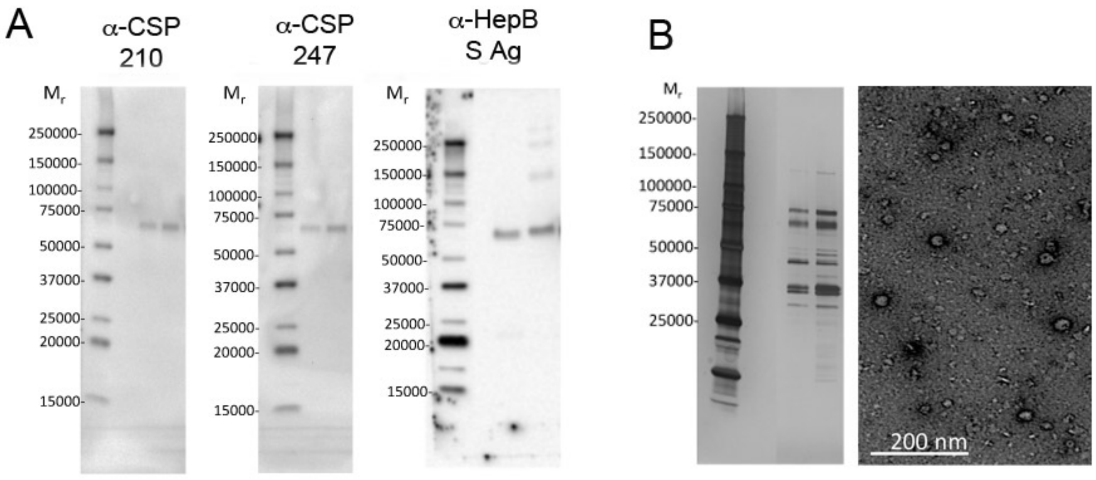

Supplementary Figure 2. immunogenicity of the Rv21 virus-like particle (VLP) presenting the PvCSPVK210/247 antigen fused to the Hepatitis B Surface antigen

(A) Anti-PvCSP VK210 antibody responses in CD-1 mice (n=6) on week 2 after VLP priming –VLP, VLP+Adj- (day 14) or on week 2 after boosting -VLP/VLP, VLP/VLP+Adj- (day 28). (B) Anti-PvCSP VK247 antibody responses in CD-1 mice (n=6) on week 2 after VLP priming –VLP, VLP+Adj- (day 14) or on week 2 after boosting -VLP/VLP, VLP/VLP+Adj- (day 28).

(C) Measurement of avidity of anti-PvCSP on the long-term with the use of Matrix M adjuvant. (D-G) Association of titers of antibody isotypes and subclasses with protection against pre-erythrocytic malaria

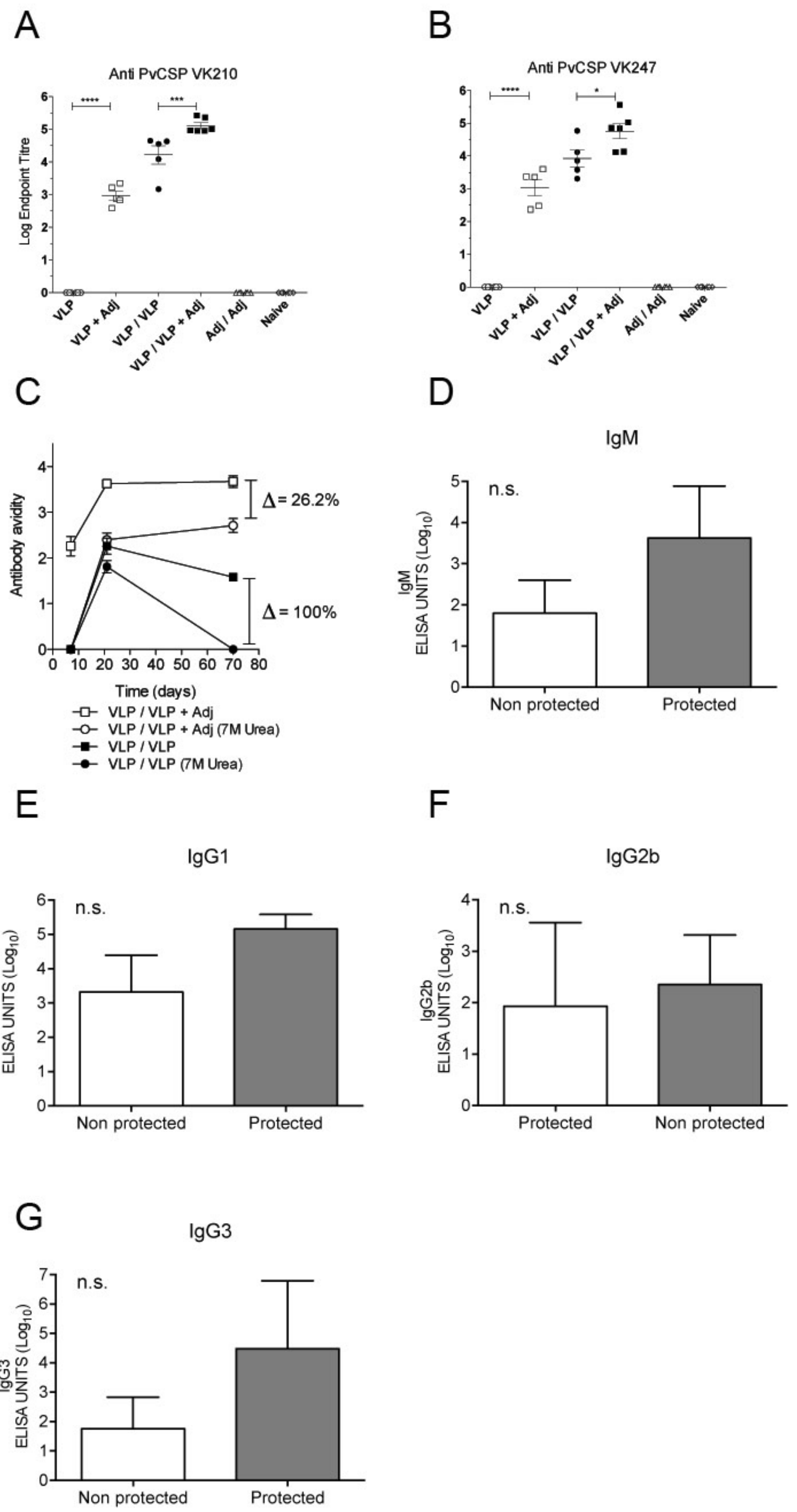

Supplement: Supplementary Information [file srep46482-s1.pdf]
